# A Further Look at Perception of Personalities in Typefaces: Evidence From Turkish

**DOI:** 10.1177/00332941241310125

**Published:** 2025-01-18

**Authors:** Mercedes Sheen, Hajar Aman Key Yekani, Ebru Morgül, Timothy R. Jordan

**Affiliations:** Department of Psychology, 150991 Heriot-Watt University, Dubai, UAE; Department of Psychology, 448243 Ibn Haldun University, Istanbul, Turkiye; School of Psychology, 199320 Canadian University Dubai, Dubai, UAE

**Keywords:** Print personality, Turkish, genderless language, semantic information

## Abstract

Previous research conducted in English indicates that the visual appearances of different typefaces are perceived as possessing distinct characteristics, what we call “print personality” (e.g., masculine, feminine, serious, fun) to the extent that the typeface used conveys information to the reader beyond that which is expressed linguistically by the word. Recent work has found that these attributions of “print personality” also extend to typefaces written in Arabic, but one language that is distinct from both languages is Turkish. Turkish is written in a version of the Latinate alphabet containing 29 letters which include unique diacritics and is a genderless language which requires no gender associations for nouns, pronouns, or adjectives. Given that many print personalities appear to be strongly associated with gender (masculine, feminine, elegance, confidence), it remains to be determined if the association of print personalities extends to Turkish typefaces, and the pattern of any such associations. Accordingly, sixteen different typefaces were presented to fluent native Turkish readers who rated each typeface according to 22 different personality characteristics. The results indicate that, although Turkish participants readily assigned personality characteristics to different typefaces, gendered associations across different typefaces were far weaker than previously found. Implications for the generality of the existence of typeface personalities across different languages, and the effect this may have on perception of genderless languages, are discussed.

## Introduction

Research indicates that the visual differences across different typefaces are associated with different personality traits, what we call *print personalities* (see Jordan et al., 2017) that provide a source of semantic information beyond what is conveyed linguistically by a word. The notion that typefaces can provide such semiotic information was raised almost a century ago by Poffenger and Franken (1923) who noted that typefaces with thin strokes were perceived as delicate and elegant while typefaces with thicker strokes were perceived as strong and independent. Over the years, the ability for typefaces to evoke non-linguistic influences on readers has been referred to in several different ways, including atmosphere value (Poffenger & Franken, 1923), geographic allusion (Lewis & Walker, 1989) and oratory effects (Mackiewicz & Moeller, 2004). Moreover, Sushan and Wright (1989) suggested that each distinctive typeface has its own personality which can be distinguished from each other in as many ways as there are typefaces. Typefaces can also become critical sources of meaning that are associated with emotions connected to a range of social, historical, and even cultural values. Spitzmüller (2012), for example, found that 
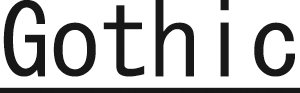
 and 
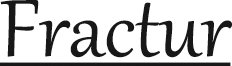
 typefaces act as markers of “Germanness”, while Haenschen and Tamul (2020) found liberal political views were more likely to be associated with messages written in sans serif typefaces. Indeed, even the presence or absence of serifs (small lines often added to the end of letter strokes) in a typeface can change the characteristics that are ascribed to a word, with typefaces containing serifs (e.g., 
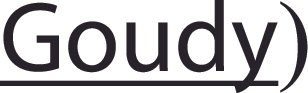
) producing more emotionally charged adjectives than sans serif typefaces (e.g., 
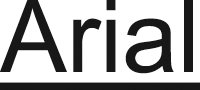
; Tantillo et al., 1995). Moreover, recent evidence from an eye-tracking study conducted in Turkiye, suggests that sans serif typefaces are read with greater speed and accuracy than typefaces with serifs (Dogusoy et al., 2016).

Observations of interactions between typefaces and linguistic meaning indicate that typefaces are not a neutral medium and can act as a powerful influence on how meanings of words are processed. For example, during a 2011 landmark moment in science, scientists at the European Organization for Nuclear Research (CERN) presented their findings concerning the Higgs boson (the God particle) in 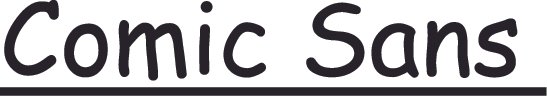, and created international uproar and concern that (presumably) such brilliant people would use a frivolous typeface for such an historical occasion (Murphy, 2017). It seems, therefore, that typefaces can undermine the importance of the words they represent in much the same way as a weak voice might fail to convey a powerful command. Indeed, evidence from more formal investigations has shown a congruity effect between a typeface and the linguistic meaning of a word (Lewis & Walker, 1989), so that when a typeface is congruent with the word it represents (e.g., the word “poetry” written in 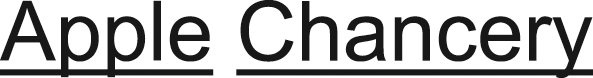), compared to when it is incongruent (e.g., the word “poetry” written in Times New Roman), words are read more fluently, reported with greater accuracy, and the message is perceived as being more genuine (e.g., Doyle & Bottomley, 2004; Lewis & Walker, 1989; Oppenheimer & Frank, 2008). Moreover, because typefaces are not a neutral conduit for conveying a message, the right typeface for the right purpose can have significant implications, not just for legibility (Zachrissen, 1965) but for other essential factors, including comprehension (Lewis and Walker), credibility (Juni & Gross, 2008; McCarthy & Mothersbaugh, 2002), fluency of perception (Oppenheimer & Frank), recall (Halamish, 2018), and even the ability to follow a recipe (Song & Schwarz, 2008).

It is generally acknowledged that, as we read, our visual system acquires different visual properties from words which provides the foundation for subsequent linguistic analyses that allows us to understand what we are reading (e.g., Allen et al., 2009; Jordan et al., 2003; Jordan et al., 2012; Jordan et al., 2016; Patching & Jordan, 2005). However, the existing evidence concerning the effects of typefaces on reading suggests that words provide more than visual information for linguistic analyses, and that the visual appearance of typefaces conveys a separate source of semantic information which is also influential when reading.

But the incidence and nature of print personalities across different languages remains to be fully explored. Research indicating that typefaces are associated with personalities (e.g., Bartram, 1982; Lewis & Walker, 1989; Poffenger & Franken, 1923) has been dominated using stimuli printed using the Latinate alphabet in English. However, more recent evidence suggests that these effects are not reliant on the use of English and that even a visually and morphologically very different language and alphabet, such as Arabic, shows evidence of the assignment of print personalities to typefaces in ways like English. Jordan et al. (2017) used Arabic typefaces common in Arabic society, and each was displayed in an Arabic sentence containing all letters of the alphabet (known as a *pangram*). Written Arabic differs substantively from English as it is read from right to left, and is written in cursive script, with very few spaces between letters and words. Moreover, the shape of each letter differs according to its position within a word, resulting in an increase in the total number of Arabic letter structures to over one hundred (see Jordan et al., 2014a; Jordan et al., 2014b). Nevertheless, Jordan et al., found that the visual structure of Arabic typefaces does indeed convey meaning (print personalities) to the extent that each of the typefaces that were used in that study generated associations of personality characteristics. The results of these findings suggest that perception of personalities in typefaces occurs across very different alphabets and is not constrained by the characteristics of just one printed language (e.g., English).

Accordingly, the focus of the present research was to explore the existence of print personalities in a language using an alphabet related to English, but which is far more recent in its evolution. The written Turkish language has a long and colorful history with the earliest Turkish alphabet dating back as far as the 7^th^ century. Since this time, the Turkish language has used several different writing structures, including Orkhon (Göktürk), Uyghur, Arabic, Cyrillic and Latin alphabets (Korkmaz, 2009). Modern day Turkish is considered a relatively new writing system, having been established in 1928 in a series of cultural reforms imposed by Ataturk, who adapted the Latin alphabet to accommodate the unique phonetic requirements of modern Turkish pronunciation. This included the addition of *ı, ü,* ö to represent the vowel sounds made in Turkish and ç, ş, j, ğ to represent consonant sounds (Korkmaz). So, although English and Turkish share the same Latin script they do not have the same alphabet and the diacritics used frequently in written Turkish may affect whether the same print personality effect is found in this new investigation of print personalities in Turkish.

Another important difference between English and Turkish (and indeed Arabic) is that the Turkish language has no morphological gender markers making it is entirely possible to read a passage of text about a person without ever being certain of their gender with the letter “o” used to denote the third person (Braun, 2001). Since the way gender in language influences perceptions of social information (e.g., Boroditsky et al., 2003) the extent to which a language discriminates between males and females is likely to guide expectations about what roles and behaviors are likely to be exhibited by men and women (Prewitt-Freilino et al., 2011). These expectations are not innate but are shaped by cultural expectations, experience, education, and beliefs that can influence, through repeated exposure, how people interpret their social environments (Bem, 1981). e.g., women are often called elegant, and men are called bold, and these frequent pairings are likely to give rise to the perception that objects considered to be elegant or bold are associated with being female or male. Calling someone an elegant or bold *person* on the other hand, would fail to produce any explicit association of that adjective to a particular gender. Thus, it seems reasonable to assume that languages which consistently distinguish between masculinity and femininity (e.g. steward, stewardess) are more likely to evoke gendered perceptions in written language and this would include the typeface in which that language is written. Indeed, evidence from English and Arabic indicates that different typefaces are readily assigned as having feminine or masculine qualities, for example, elegant, warm, confident, serious (Bhatia & Bhatia, 2021; Seitchik, 2020) to the extent that typefaces that were rated the highest in masculinity were also rated the lowest in femininity (Jordan et al., 2017). The consistency with which gender is associated to typefaces is likely to arise because typefaces activate associations with the situation in which different typefaces are encountered regularly. Thus, if the association of print personalities to different typefaces is influenced by having gender as a grammatical category, it remains to be determined whether typefaces in a genderless language such as Turkish will elicit the same perception of print personalities to the extent previously found using Arabic. Indeed, since Arabic is a gendered language and is visually and morphologically very different to modern-day Turkish, such a finding would add to the growing body of literature and indicate that the association of print personalities to different typefaces is a general aspect of language perception across languages with different writing systems and in different cultural contexts.

Accordingly, this study is the first to investigate whether fluent Turkish readers assign different print personalities to different Turkish typefaces. If this attribution was found, it would provide strong evidence that the assignment of print personalities to typefaces is a common aspect of language perception, with significant implications for understanding how written language is processed.

## Method

### Ethics Statement

In accordance with the Declaration of Helsinki, this study received ethical approval from a University in Istanbul, Tukiye, and informed consent was obtained from all participants.

### Participants

Participants were invited to take part through advertising around the university. Participants were invited to take part through advertising around the university. Our main criterion for inclusion was fluent reading of Turkish text by native Turkish readers. Accordingly, all participants were screened for their ability to read Turkish, and this included reading rates as well as vocabulary tests (see Patching & Jordan, 2005) and those selected were chosen because they satisfied the requirements for fluent Turkish reading ability and satisfied additional criteria for visual ability (clearly, the experiment depended on visual perception). Following this procedure, sixty-four participants (32 females) with an age range of 18–26 (*m* = 21.84 and *SD* = 2.23) participated in the experiment. All participants had normal, or corrected-to-normal vision, as determined by Baily-Lovie (Baily & Lovie, 1980) and Pelli-Robson et al. (1988) assessments (see Jordan et al., 2011).

### Stimuli

Sixteen Turkish typefaces (see Table 1) used commonly in Turkish society were each presented in a pangram (a sentence containing all letters of the alphabet) with each typeface presented in 14-point. We used a pangram common to Turkish which translates as: *The patient in pajamas quickly trusted the swarthy driver*). Participants were asked to rate how much they associated each of the 22 different personality traits for each different typeface, using a 1-7 scale (1 = *not at all*, 7 = *very*). The personality traits used in this study were: cheap, cold, cruel, confident, dignified, elegant, feminine, formal, friendly, inviting, kind, loud, masculine, playful, pretentious, professional, relaxed, scholarly, serious, sloppy, straight and warm and were chosen based on formative work on the persona of typefaces conducted by Brumberger (2003).

**Table 1.** The Typefaces Used in This Study.

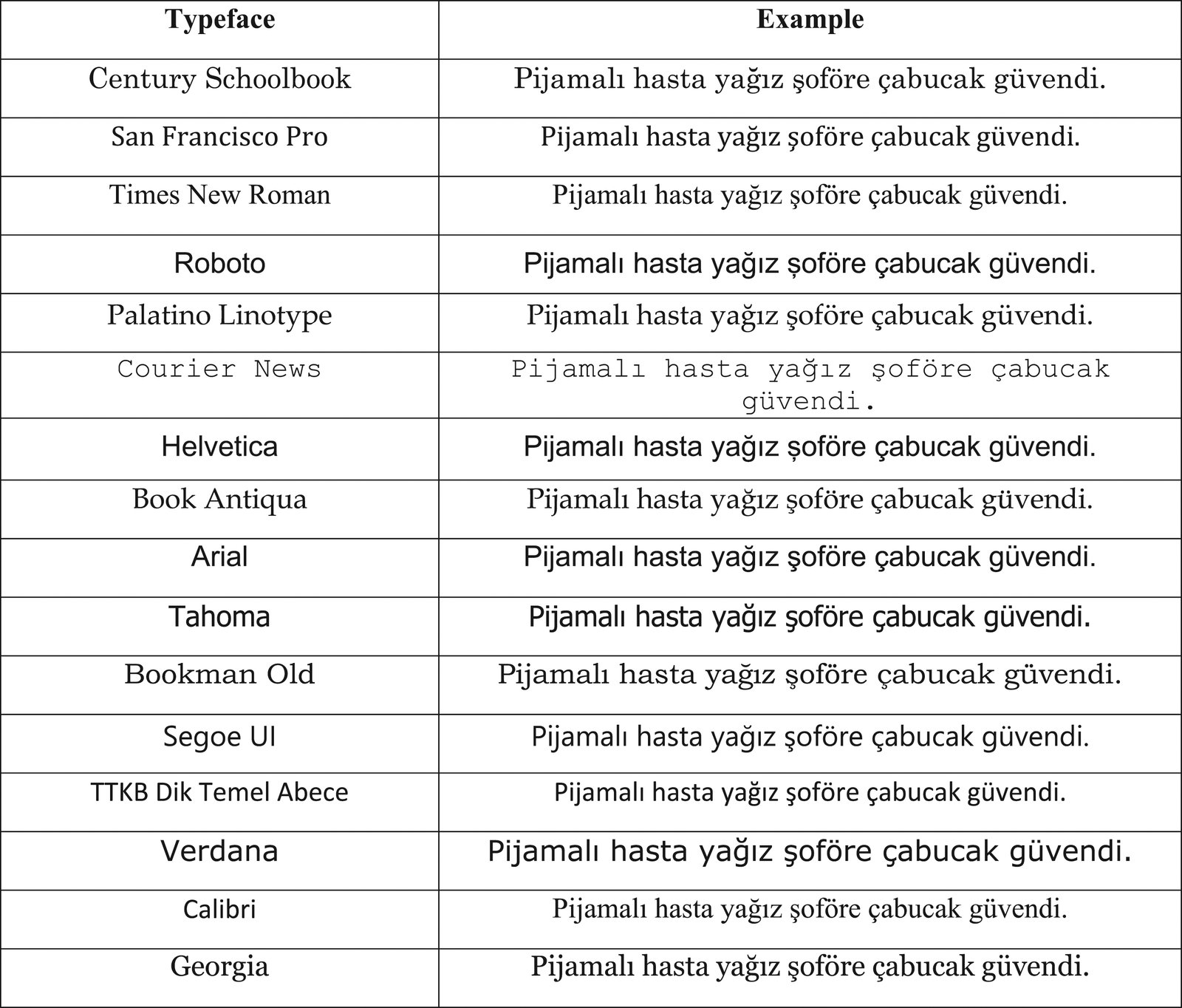

### Design & Procedure

We used the same experimental procedure as we did previously with Arabic typefaces (see Jordan et al., 2017). Turkish typeface was a within-participant independent variable and ratings of the personality characteristics was the dependent variable. Each participant took part individually in a quiet room in the psychology laboratory and was given a booklet containing sixteen separate sheets of paper with each sheet containing the pangram in one of the sixteen different Turkish typefaces. Each sheet also contained the list of twenty-two personality characteristics which were listed in reverse order for half of the participants to counteract effects of response bias and fatigue. The sheets in each booklet were arranged in a different random order. Instructions were given in Turkish orally to participants who were asked to make their ratings carefully. Each participant took approximately 20 minutes to complete the task.

## Results

For each typeface the mean rating (*M*) for each personality characteristic and the standard deviation of these ratings (*SD*) are shown in Table 2. For each personality characteristic, a repeated-measure one-way analysis of variance (ANOVA) with the factor of typeface was conducted to determine whether each characteristic was rated differently across different typefaces. The results of these analyses are shown in Table 3. Subsequent pair-wise comparisons between typefaces for each personality characteristic were performed using Bonferroni-corrected *t-*tests.

**Table 2.** For Each Typeface, Mean Rating (*M*) and Standard Deviation (*SD*) are Shown for Each Personality Characteristics.

Typeface		Cheap	Cold	Confident	Cruel	Dignified	Elegant	Feminine	Formal	Friendly	Inviting	Kind	Loud	Masculine	Playful	Pretentious	Professional	Relaxed	Scholarly	Serious	Sloppy	Straightforward	Warm
Century schoolbook	M	1.9	2.6	4.5	1.8	3.3	3.7	2.7	3.5	3.3	3.7	3.7	1.9	2.6	2.2	2.5	4.2	3.6	3.4	3.6	1.9	2.8	3.3
	SD	1.4	1.8	1.7	1.3	1.5	1.8	1.9	2.0	1.8	1.9	1.9	1.2	1.6	1.6	1.5	1.9	1.8	1.4	1.8	1.4	1.6	1.8
San Francisco	M	2.9	4.2	3.9	3.4	2.9	1.6	1.9	3.2	2.2	1.7	1.7	4.5	3.0	2.6	3.8	2.7	2.6	2.1	3.8	2.7	3.2	2.2
	SD	2.0	2.0	2.0	1.9	2.1	1.2	1.0	2.1	1.5	1.3	1.3	2.5	2.1	2.1	2.1	1.8	2.1	1.5	2.2	1.8	2.1	1.6
Times new Roman	M	1.8	3.0	4.1	2.1	3.0	3.4	2.7	3.8	3.0	3.2	3.5	1.9	2.0	2.1	2.8	4.2	3.4	3.2	3.9	2.0	2.5	3.2
	SD	1.2	1.8	1.7	1.5	1.5	1.5	1.7	1.7	1.7	1.6	1.5	1.4	1.3	1.3	1.8	1.7	2.0	1.5	2.0	1.7	1.6	1.7
Roboto	M	1.6	3.4	3.4	2.4	2.9	3.0	2.1	3.3	3.3	2.5	3.0	1.8	2.4	2.1	2.6	3.4	3.9	2.7	3.2	2.6	4.1	3.2
	SD	1.0	2.3	1.8	1.8	1.6	1.9	1.4	2.1	1.8	1.7	1.8	1.1	1.7	1.4	1.7	2.0	2.0	1.4	2.0	1.8	2.1	1.8
Palatino linotype	M	1.7	2.4	3.8	1.9	3.2	3.6	2.5	3.9	3.3	3.5	3.4	2.1	2.5	2.6	2.2	4.0	3.8	3.3	3.8	2.3	3.7	3.3
	SD	1.2	1.4	1.5	1.2	1.8	1.7	1.7	2.0	1.7	1.8	1.7	1.4	1.5	1.8	1.4	1.9	2.0	1.7	1.7	1.5	1.8	1.6
	SD	2.0	2.3	2.0	2.2	2.0	2.2	1.4	2.4	1.8	2.2	2.1	2.1	1.7	1.8	2.0	2.2	2.0	1.8	1.9	2.0	2.1	2.0
Helvetica	M	3.4	4.1	3.9	4.2	2.5	1.4	1.5	2.4	1.9	1.6	1.5	5.1	3.2	2.8	4.2	2.0	1.9	1.8	3.7	2.9	2.9	1.9
	SD	2.1	2.3	2.2	2.1	2.0	0.9	0.9	2.0	1.4	1.2	1.3	2.3	1.9	2.2	2.3	1.5	1.6	1.3	2.2	2.0	2.0	1.4
Book antiqua	M	1.6	2.7	4.4	2.0	3.5	3.9	2.8	3.6	3.3	3.6	3.7	2.1	2.3	2.8	2.8	4.0	3.3	3.3	3.7	1.8	3.2	3.4
	SD	1.1	1.9	1.7	1.5	1.8	1.8	1.8	1.9	1.9	1.7	1.8	1.2	1.5	1.8	1.7	2.0	1.9	1.8	2.0	1.5	1.9	1.9
Arial	M	1.8	3.2	3.3	2.2	3.4	3.1	1.9	3.5	3.3	2.4	3.3	2.0	2.3	2.2	2.7	3.5	3.6	3.2	3.5	1.9	4.2	3.3
	SD	1.4	1.9	1.6	1.6	1.9	1.5	1.2	2.1	1.8	1.2	1.8	1.5	1.5	1.3	1.8	1.7	2.0	1.8	1.9	1.4	2.1	1.7
Tahoma	M	2.1	3.3	3.6	2.4	2.9	2.4	1.6	2.9	2.8	2.6	2.8	2.4	2.6	2.6	3.0	3.1	3.7	2.7	3.4	2.5	3.6	2.7
	SD	1.4	2.0	1.8	1.7	1.7	1.5	0.9	2.0	1.7	1.4	1.4	1.6	1.5	1.9	2.0	1.8	2.0	1.6	1.9	1.7	1.7	1.5
Bookman old	M	2.1	3.2	4.1	2.3	3.3	3.2	2.6	3.2	2.7	3.2	3.4	2.0	2.3	2.5	3.1	3.5	3.3	3.2	3.6	2.3	3.2	2.9
	SD	1.4	1.9	1.8	1.7	1.8	1.8	1.8	1.9	1.4	1.8	1.9	1.4	1.5	1.3	1.8	1.9	1.6	1.8	1.9	1.5	1.8	1.7
Segoe UI	M	2.2	3.2	3.3	2.2	3.1	2.6	2.0	3.1	3.3	2.4	3.0	1.6	2.5	2.2	2.5	3.5	3.8	3.0	3.0	1.9	3.9	3.3
	SD	1.8	2.1	1.4	1.5	1.4	1.4	1.4	1.9	1.6	1.3	1.7	1.2	1.6	1.5	1.9	1.9	1.8	1.5	1.8	1.3	2.0	1.7
TTKB	M	1.6	2.1	3.0	1.5	1.9	3.4	2.5	1.5	4.4	3.3	3.7	2.7	1.7	3.6	2.9	2.0	4.4	2.1	1.6	2.3	3.4	4.3
	SD	1.1	1.5	1.8	1.1	1.2	2.2	1.9	1.1	1.9	2.1	2.0	1.9	1.0	2.2	1.7	1.5	2.0	1.3	0.9	1.5	2.0	2.0
Verdana	M	2.1	3.3	3.4	2.1	2.6	2.6	2.0	2.9	3.2	2.5	2.9	2.7	2.5	2.5	3.1	2.9	3.9	2.8	2.8	3.1	3.8	3.1
	SD	1.6	2.2	1.6	1.6	1.4	1.5	1.4	1.9	2.0	1.6	1.6	2.0	1.5	1.6	1.9	1.7	1.9	1.6	1.7	2.0	2.1	1.7
Calibri	M	1.7	2.5	3.4	2.2	3.3	3.2	2.1	3.8	3.1	3.1	3.6	1.8	2.2	2.2	2.2	3.8	3.3	3.4	3.3	1.8	3.8	3.4
	SD	1.4	1.8	1.6	1.6	1.8	1.9	1.3	2.1	1.9	2.0	1.8	1.1	1.3	1.5	1.4	2.0	1.7	1.8	2.1	1.2	2.1	1.7
Georgia	M	1.7	3.0	4.1	2.1	3.0	3.6	2.7	3.6	3.3	3.1	3.7	2.0	2.1	2.2	2.3	4.2	3.2	3.6	3.5	1.8	2.8	3.6
	SD	1.2	1.9	1.7	1.4	1.7	1.7	2.1	1.9	1.7	1.6	1.7	1.4	1.3	1.5	1.4	1.5	2.0	1.7	2.0	1.5	1.8	1.7

**Table 3.** Results of Analyses of Variance Comparing the Ratings of Each Personality Characteristic Across the Typefaces.

Personality characteristic	*Df* Typeface	*df*Error	*F*	*p* =	$\eta^{2}$
Cheap^***^	7.14	228.41	5.58	0.000	0.15
Cold^***^	15.00	480.00	3.70	0.000	0.10
Confident^**^	9.24	295.82	3.28	0.001	0.09
Cruel^***^	8.46	270.66	8.21	0.000	0.20
Dignified^*^	9.20	294.35	2.42	0.011	0.07
Elegant^***^	8.68	277.66	7.79	0.000	0.20
Feminine^***^	7.55	241.66	5.12	0.000	0.14
Formal^***^	8.77	280.55	5.46	0.000	0.15
Friendly^***^	8.81	282.02	5.55	0.000	0.15
Inviting^***^	8.29	265.44	6.50	0.000	0.17
Kind^***^	9.04	289.19	6.84	0.000	0.18
Loud^***^	7.92	253.47	13.02	0.000	0.29
Masculine^**^	6.15	196.88	3.21	0.005	0.09
Playful^*^	8.86	283.47	2.16	0.025	0.06
Pretentious^***^	9.21	294.75	3.79	0.000	0.11
Professional^***^	8.49	271.66	8.22	0.000	0.20
Relaxed^***^	9.66	309.10	4.29	0.000	0.12
Scholarly^***^	8.52	272.75	5.25	0.000	0.14
Serious^***^	8.42	269.34	4.87	0.000	0.13
Sloppy^**^	8.24	263.62	2.90	0.004	0.08
Straightforward^**^	8.44	270.21	3.34	0.001	0.09
Warm^***^	8.79	281.38	5.33	0.000	0.14

Degrees of freedom are Greenhouse-Geisser corrected where appropriate.

*significant at *p* < .05 level, **significant at *p* < .01 level, ***significant at *p* < .001.

As indicated in Table 4 (see also Figure 1), typefaces produced widespread effects on the ratings of each of the 22 personality characteristics and these effects were examined more closely using pair-wise Bonferroni-corrected *t-*tests to determine precisely, for each personality characteristic. For example, *Palatino Linotype* was rated as more elegant 3.6 than *San Francisco Pro* 1.6 (*p < 0.001*) and *Helvetica Neue* 1.4 (*p < 0.001*); whilst *TTKB* was rated as less professional 2.0 compared to *Century Schoolbook* 4.2, *Times New Roman* 4.2 and *Georgia* 4.2 (*p*s *< 0.001*).

**Figure 1.**
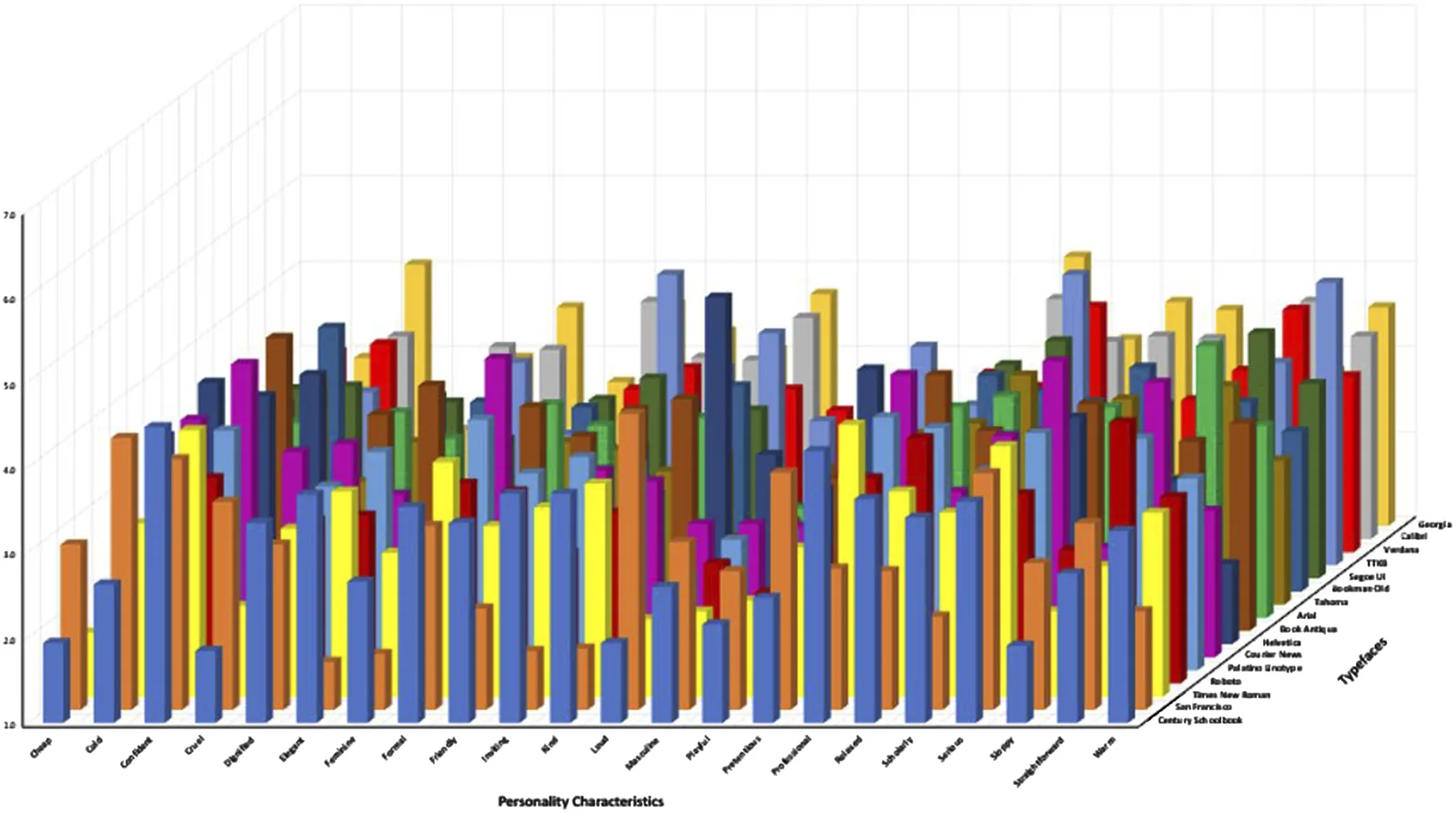
Association between typefaces and personality characteristics.

### Frequency-Based Grouping Criterion

To unify the findings and identify overarching factors applicable across all typefaces, a frequency-based criterion was employed. Specifically, personality traits that clustered together in more than half (i.e., at least 8 out of 16) of the typefaces were retained as factors. This frequency threshold ensured that only consistent and meaningful patterns were included in the final factor set. For example, traits such as “warm,” “inviting,” and “friendly” were grouped in most of the typefaces, thus forming one of the final factors.

### Internal Consistency Check with Cronbach’s Alpha

To assess the reliability of the identified factors, Cronbach’s alpha was calculated for the relevant factors (factors 1–4). Factors that consisted of single items (e.g., Playful, Sloppy, Cold, Confident, and Straightforward) did not require Cronbach’s alpha, as this measure is not applicable for individual items. This step was performed to evaluate the internal consistency and ensure that the traits within each factor were cohesively related.

### Masculinity Versus Femininity

In this study, a series of paired-samples t-tests were conducted to compare participants’ ratings of 16 different typefaces in terms of perceived masculinity and femininity. The results showed that for 11 of the typefaces, participants rated masculinity and femininity similarly, indicating no significant differences between the two traits. However, for five typefaces, significant differences were found between masculinity and femininity ratings. Additionally, a series of Spearman’s correlation analyses were performed to assess the relationship between the two personality traits (masculinity and femininity) across the typefaces. The results demonstrated a positive correlation in 13 of the typefaces, suggesting a consistent relationship between how these traits were perceived.

For detailed statistical results, please refer to Tables 4 and 5, which presents the means, standard deviations, t-values, and significance levels for the paired-samples t-tests, as well as the correlation coefficients for the typeface ratings Tables 6–10.

**Table 4.** Co-Occurrence Frequency of Personality Traits (Elegant, Friendly, Kind, Warm, Inviting, Relaxed) Across Typefaces for Factor 1.

Factor 1	Elegant	Friendly	Kind	Warm	Relaxed	Inviting
Friendly	14	0	14	15	11	13
Kind	16	14	0	13	9	15
Warm	13	15	13	0	12	11
Relaxed	9	11	9	12	0	8
Inviting	15	13	15	11	8	0

**Table 5.** Co-occurrence Frequency of Personality Traits (Serious, Formal, Dignified, Professional, Scholarly) Across Typefaces for Factor 2.

Factor 2	Serious	Formal	Dignified	Professional	Scholarly
Formal	15	0	11	16	13
Dignified	13	11	0	16	10
Professional	15	16	12	0	14
Scholarly	12	13	10	14	0

**Table 6.** Co-Occurrence Frequency of Personality Traits (Loud, Pretentious, Cheap, Cruel) Across Typefaces for Factor 3.

Factor 3	Loud	Pretentious	Cheap	Cruel
Pretentious	10	0	9	9
Cheap	11	9	0	14
Cruel	10	9	14	0

**Table 7.** Co-Occurrence Frequency of Personality Traits (Masculine, Feminine) Across Typefaces for Factor 4.

Factor 4	Masculine
Feminine	11

**Table 8.** Cronbach’s Alpha Scores for Factors 1-4 Across 16 Typefaces.

Factor	Measurement models	Number of items	Threshold	Cronbach’s alpha
Factor 1 Friendly Kind Warm Relaxed Inviting	Century schoolbook	6	.60	.87
	San Francisco	6	.60	.83
	Times new roman	6	.60	.88
	Roboto	6	.60	.84
	Palatino linotype	6	.60	.85
	Courier news	6	.60	.80
	Helvetica	6	.60	.87
	Book antiqua	6	.60	.92
	Arial	6	.60	.87
	Tahoma	6	.60	.83
	Bookman old	6	.60	.81
	Segoe UI	6	.60	.81
	TTKB	6	.60	.86
	Verdana	6	.60	.86
	Calibri	6	.60	.91
	Georgia	6	.60	.87
Factor 2 Formal Dignified Professional Scholarly	Century schoolbook	5	.60	.74
	San Francisco	5	.60	.75
	Times new roman	5	.60	.83
	Roboto	5	.60	.84
	Palatino linotype	5	.60	.85
	Courier news	5	.60	.88
	Helvetica	5	.60	.77
	Book antiqua	5	.60	.86
	Arial	5	.60	.84
	Tahoma	5	.60	.85
	Bookman old	5	.60	.88
	Segoe UI	5	.60	.78
	TTKB	5	.60	.82
	Verdana	5	.60	.83
	Calibri	5	.60	.85
	Georgia	5	.60	.80
Factor 3 Pretentious Cheap Cruel	Century schoolbook	4	.60	.72
	San Francisco	4	.60	.78
	Times new roman	4	.60	.74
	Roboto	4	.60	.63
	Palatino linotype	4	.60	.63
	Courier news	4	.60	.62
	Helvetica	4	.60	.72
	Book antiqua	4	.60	.72
	Arial	4	.60	.69
	Tahoma	4	.60	.72
	Bookman old	4	.60	.76
	Segoe UI	4	.60	.76
	TTKB	4	.60	.67
	Verdana	4	.60	.72
	Calibri	4	.60	.47
	Georgia	4	.60	.77

**Table 9.** Paired-Sample t Test Results for Masculinity and Femininity Ratings Across 16 Typefaces.

Typefaces	M	SD	t	df	p
Century schoolbook	0.14	2.27	0.50	63	0.62
San Francisco***	1.56	2.45	5.10	63	0.00
Times new roman*	−0.64	2.13	−2.39	63	0.02
Roboto	0.19	1.50	1.00	63	0.32
Palatino linotype	0.23	2.01	0.93	63	0.36
Courier news	0.38	2.17	1.38	63	0.17
Helvetica***	1.63	2.36	5.51	63	0.00
Book antiqua	−0.34	2.06	−1.34	63	0.19
Arial	0.02	1.64	0.08	63	0.94
Tahoma*	0.64	1.95	2.62	63	0.01
Bookman old	−0.05	1.90	−0.20	63	0.84
Segoe UI	0.19	1.58	0.95	63	0.35
TTKB***	−1.02	1.99	−4.09	63	0.00
Verdana	0.28	1.63	1.38	63	0.17
Calibri	−0.20	1.50	−1.08	63	0.28
Georgia	−0.14	1.97	−0.57	63	0.57

**Table 10.** Spearman’s Correlation Matrix for Masculinity and Femininity Ratings Across 16 Typefaces.

Typeface	*P* (Masculinity-Femininity)	*p*
Century schoolbook	.25^*^	0.047
San Francisco	0.19	0.130
Times new roman	.43^***^	0.000
Roboto	.61^***^	0.000
Palatino linotype	.41^**^	0.001
Courier news	0.22	0.075
Helvetica	0.17	0.179
Book antiqua	.35^**^	0.004
Arial	.53^***^	0.000
Tahoma	.32^**^	0.009
Bookman old	.42^**^	0.001
Segoe UI	.58^***^	0.000
TTKB	.34^**^	0.006
Verdana	.57^***^	0.000
Calibri	.65^***^	0.000
Georgia	.54^***^	0.000

*Note.* ρ = Spearman’s correlation coefficient.

## Discussion

The aim of this research was to establish whether fluent Turkish readers associated different print personalities to Turkish typefaces. Earlier research in this area had so far only used English and Arabic stimuli and so the existence of this phenomenon in other languages, especially one using an adapted Latinate alphabet, was not known. The results clearly indicate that, just as previously found in Arabic with native Arabic readers (Jordan et al., 2017), the visual make-up of Turkish typefaces does indeed convey meaning beyond linguistics in native Turkish readers. Moreover, these results add to the growing body of literature by offering new and important indications as to how common the phenomenon of attributing personality characteristics to typefaces is across different language structures and cultural contexts.

Accordingly, despite written Turkish being a relatively new language, the visual attributes of different Turkish typefaces influence the perception of words apart from matters of legibility (Kostelnick, 1990). Each of the Turkish typefaces used in this study were rated as having distinct print personalities, but how does the visual structure of a typeface generate specific personality associations. It is likely that readers regularly come across typefaces in certain circumstances (e.g., government documents, formal invitations, places of worship) and these associations determine the print personality attributed to that typeface. Moreover, the consistency with which participants attributed personality characteristics across the different typefaces supports the notion that perceptions are influenced by prior experience and by associations connected to those experiences which provide routes for learning and remembering (Bandura & Rosenthal, 1966). Indeed, one example from the study reported here were the ratings for Times New Roman. In 2004, the Turkish government decreed that all official documentation is written in Times New Roman and, given their age, the participants who took part in our study would only ever have encountered Times New Roman in government documents and this was reflected in the consistent ratings for “confident” 4.1, “formal” 3.8, “scholarly” 3.9 and conversely “cheap” 1.8 and “loud” 2.0.

Another explanation for the way in which print personalities are formed may be linked to the attribution of human traits to inanimate objects. For example, extant evidence indicates that beliefs about associations with gender may be elicited by the physical attributes of non-social objects such that, when activated, associations of gender may still influence subsequent beliefs about that object without any conscious intentions to do so. Indeed, gender has been associated to many non-social items including furniture (Gal & Wilkie, 2010), numbers (Wilkie & Bodenhausen, 2012; Jordan et al., 2017), food (Wilkie & Bodenhausen, 2015), brands (Neale et al., 2016) and even simple shapes (Stroessner et al., 2020). Similar associations were made by our participants who rated typefaces as being different from each other across several other personality characteristics. For example, the ratings for the typeface TTKB (see Table 1), which was mandated by the Ministry of National Education for use in all audio-visual literacy training from 2017 onwards, were consistent with this directive with average ratings for “friendly” and “relaxed” of 4.4 and “formal” and “serious” 1.6. Similarly, Helvetica (see Table 1) had the highest rating for “cold” and the lowest rating for “friendly”, whilst Georgia had one of the highest ratings for “confident” and the lowest for “cheap”.

In addition, despite Turkish being a genderless language, gender remains a salient social construct in Turkiye and has a significant influence on patterns of behavior in many spheres of daily life (Braun, 2001). For instance, according to Turkish Civil Law, women and men are afforded different rights and obligations in marriage, and there are clear ideas about what characteristics, pursuits, and occupations are appropriate for males and females. However, if few gender distinctions in written Turkish exist, how is gender communicated and perceived, and what role does written language play in the social construction of gender. Interestingly, although the Turkish typefaces were consistently rated as either masculine or feminine, the differences in gender ratings were relatively weak, particularly when compared to our previous findings for Arabic typefaces (a gendered language). Indeed, of the sixteen typefaces, only five of the typefaces differed significantly in their ratings on the “masculine” and “feminine” scales. One explanation for the weak gender associations to typefaces may be that the way in which we perceive masculinity and femininity is, in part, shaped by how we write about them. Thus, the absence of grammatical gender on the cognitive representations of men and women is likely to shape how people construe their social worlds along gender lines. Evidence for this assertion comes from a study by Chen and Su (2011), who examined whether the use gender in English, and the lack of gender in Chinese, affected the way participants answered gender-related questions after reading the same passage of text in their own language. Their results showed that English participants responded more quickly and with greater accuracy than Chinese participants in answering gender-related questions associated to the text. Given this, and other recent research linking gendered perceptions of the social world to language (e.g., Boroditsky et al., 2003) one could argue that when a language rarely discriminates between gender, individuals speaking and reading that language will be less likely to use gender to organize their beliefs around gender and this could help in explaining the results reported here.

The objective of the research reported here was to determine whether perception of print personalities would extend to a language that uses an adapted Latinate alphabet and has no morphological gender by fluent readers of modern-day Turkish. Moreover, despite the possibility that our participants may not have encountered each of the sixteen of the typefaces used in the study, our results show the visual characteristics of different Turkish typefaces elicit perception of print personalities independent of their linguistic meaning. Although this was the first study to look at Turkish print personalities, we provide strong evidence that using the proper typeface for the proper purpose may have the same consequences for matters such as comprehension, readability and perceptual fluency for Turkish as they do in English and Arabic. Moreover, our research supports the view that perception of typefaces provides a critical source of meaning and is, as our work indicates, a general component of language perception.

## Data Availability

The data that support the findings of this study are available from the corresponding author upon request.*
